# Spine imaging after lumbar disc replacement: pitfalls and current recommendations

**DOI:** 10.1186/1754-9493-3-15

**Published:** 2009-07-20

**Authors:** Yohan Robinson, Bengt Sandén

**Affiliations:** 1 Uppsala University Hospital, Institute for Surgical Sciences, Department of Orthopaedics, Uppsala, Sweden

## Abstract

**Background:**

Most lumbar artificial discs are still composed of stainless steel alloys, which prevents adequate postoperative diagnostic imaging of the operated region when using magnetic resonance imaging (MRI). Thus patients with postoperative radicular symptoms or claudication after stainless steel implants often require alternative diagnostic procedures.

**Methods:**

Possible complications of lumbar total disc replacement (TDR) are reviewed from the available literature and imaging recommendations given with regard to implant type. Two illustrative cases are presented in figures.

**Results:**

Access-related complications, infections, implant wear, loosening or fracture, polyethylene inlay dislodgement, facet joint hypertrophy, central stenosis, and ankylosis of the operated segment can be visualised both in titanium and stainless steel implants, but require different imaging modalities due to magnetic artifacts in MRI.

**Conclusion:**

Alternative radiographic procedures should be considered when evaluating patients following TDR. Postoperative complications following lumbar TDR including spinal stenosis causing radiculopathy and implant loosening can be visualised by myelography and radionucleotide techniques as an adjunct to plain film radiographs. Even in the presence of massive stainless steel TDR implants lumbar radicular stenosis and implant loosening can be visualised if myelography and radionuclide techniques are applied.

## Background

Total disc replacement (TDR) is an evolving surgical option for patients with degenerative disc disease [1,2]. The appearance of disc arthroplasty has required radiologists to contend with a new entity of spinal implants. Many fusion devices are made of titanium or carbon, howewer the currently available lumbar TDR-implants are made of stainless steel, which obscures imaging when using MR-diagnostics (Table 1).

**Table 1:** Alloy and articulation specifics of available lumbar TDR implants

**Implant name**	**Charité III**	**ProDisc**	**Maverick**	**Activ L**
Manufacturer	DePuy	Synthes	Medtronic	B. Braun

Alloy	CoCr	CoCr	CoCr	CoCr

Articulation	Non-constrained PE inlay	Semi-constrained PE-inlay	Metal-metal	Semi-constrained PE-inlay

It is widely accepted that lumbar disc arthroplasty is associated with several access- and implant-associated complications. Unfortunately imaging modalities after TDR cannot simply be transferred from fusion patients. In this review the complications following total disc replacement are discussed and guidelines for imaging following lumbar disc arthroplasty are provided.

### Imaging modalities after lumbar total disc replacement

All commercially available lumbar artifical discs are composed of a stainless steel core with titanium surfacing (Table 2). This has implications on the accuracy of postoperative magnetic resonance (MR) diagnostics. Sekhon et al [3] compared the diagnostic quality of MR-imaging with regard to detection of central and foraminal spinal stenosis after cervical disc replacement. They found that only titanium devices allow a proper postoperative visualisation of neural structures at the operated and adjacent levels. Matsuura et al [4] investigated the artifact size of different metals/alloys in MR imaging and found zirconia and aluminium to cause the least artifacts, followed by pure titanium and titanium alloys. Greatest artifacts were caused by stainless steel. Fast-spin echo sequences with lower voxel-size may minimise the artefacts, but stainless steel still has an incomparably worse effect on image quality [5].

**Table 2:** Imaging guidelines after lumbar total disc replacement with regard to implant alloy

**Complication/Alloy**	**Titanium**	**Stainless steel (CoCr)**
Metallosis/PE wear loosening	Plain x-ray, scintigraphy, PET-CT	Plain x-ray, scintigraphy, PET-CT

Implant failure/fracture	Plain x-ray, CT	Plain x-ray, CT

Implant/Core dislocation	Plain x-ray, CT	Plain x-ray, CT

Infection	MR, PET-CT, radiolabelled white blood cell scintigraphy	PET-CT, radiolabelled white blood cell scintigraphy

Late facet degeneration/secondary stenosis	MR, CT	Myelography, CT

Implant malpositioning	Plain x-ray, CT	Plain x-ray, CT

Implant subsidence	Plain x-ray, CT	Plain x-ray, CT

Segmental fusion	CT, dynamic plain x-ray	CT, dynamic plain x-ray

First-line diagnostics appear first.

Myelography alone and later combined with CT has for many years been the primary diagnostic means in suspected lumbar spinal stenosis. Myelographic findings in central canal stenosis include complete or partial block to the contrast column, appearing as an hour-glass constriction on the AP view. There appears to be poor correlation between clinical symptoms and findings at myelography, with myelography commonly showing more abnormalities than would be expected [6,7], although some authors found a block to the flow of contrast medium to be a good predictor of successful outcome following spinal decompression [8].

Scintigraphy with 99mTc radionuclide tagged white blood cells can be a helpful means in diagnosing suspicious infections [9]. 99mTc-hexamethylpropylene amine oxime (HMPAO) is generally more sensitive for the detection of acute osteomyelitis than of chronic osteomyelitis (with a high sensitivity 97.7% and a specificity of 96.8%) [10]. 99mTc-HMPAO is also able to separate septic from aseptic bone lesions [11]. Leukocyte imaging can be applied to chronic osteomyelitis (including infected joint prostheses). 111In-Oxine is preferred over 99mTc-HMPAO in such a case because it can be performed simultaneously with 99mTc-stannous fluoride colloid scanning for the assessment of bone marrow involvement (mismatched defects identify infectious foci). Infected orthopaedic hardware will have increased uptake of 111In-oxine, whereas activity will be normal or decreased in healthy or loose prostheses [10].

Postoperative low-grade-infection or implant loosening can be hard to visualise. As in hip or knee arthroplasty radionuclide scintigraphy has some value with regard to sensitivity, but has lower specificity with regard to loosening or low-grade infection. Gallium 67 scans have a sensitivity and specificity of 89% and 85% [12].

In the last years the increased availability of positron emission tomography (PET) enabled postoperative diagnostics with greater specificity for infection. Gratz et al [13] found fluorine-18 fluorodeoxyglucose (FDG) hybrid PET superior to MRI, 67Ga citrate and 99mTc-methylene diphosphonate (MDP), especially in patients with low-grade spondylitis, adjacent soft tissue infections and advanced bone degeneration. To visualise increased bone turnover 18F-flouride-PET is an option, and found helpful to diagnose aseptic artificial knee joint loosening [14]. There is no clinical evidence available with regard to PET after TDR, but is very likely that this imaging modality will emerge to a more specific diagnostic tool and replace 99mTc-MDP scintigraphy in cases with suspected infections or implant loosening where MRI is obsolete [15].

### Indication and options for imaging after lumbar total disc replacement

Despite the biomechanically appealing concept of motion preservation, lumbar disc arthroplasty is associated with access-related and implant-specific complications. Access and implant related complications can be due to technical errors or may be due to abnormal anatomy [16]. Implant wear and loosening are known to occur in all types of arthroplasty.

#### a) Access-related complications

All commercially available FDA-approved lumbar total disc replacements require an anterior access for the implantation of artificial discs. Lumbar discs can be implanted via a transperitoneal or a retroperitoneal approach, of which the latter is favoured by most surgeons. Complications with regard to these accesses are well known and differ not from those of anterior fusion, even though TDR-implantation requires a wider exposure of the disc, which often requires more traction and pressure of retractors on the surrounding tissue [17]. Especially revision-surgery is associated with higher morbidity and mortality compared to the index surgery [18]. Revision may require a transperitoneal approach if the index intervention was retroperitoneal. Then vascular and ureteric stenting may reduce the risk for access-related complications. Nevertheless the availability of a vascular surgeon on call is highly recommended in revision cases [19].

Most access-related complications can be visualised by sonography and abdominal CT with intravenous contrast. In few cases an angiography or an uretherography may be necessary.

#### b) Infections

There is no specific data on the occurrence of infection of artificial discs, but early and mid-term infections may occur as likely as in anterior fusion with 0 and 12% in lumbar instrumented fusion [20]. Nevertheless due to the prevalence of motion and a neo-joint formation, late haematogenic spread infection as known from hip and knee arthoplasty is possible in the immunocompromised TDR patient, with detrimental effects on the patient's health and function.

Postoperative infections of the spine can optimally be visualised by MR with contrast. A sensitivity and specificity of 93% and 97% makes the MRI superior to all other radiological imaging modalities [21]. If MRI-scans are not available or stainless steel implants were used radionuclide imaging should be used to reveal focal increased uptake. If available PET-CT scans should be considered to diagnose a suspicious postoperative spinal infection, since PET has a higher specificity than radionuclide scintigraphy.

#### c) Implant dislocation and polyethylene inlay dislodgment

**Figure 1:**
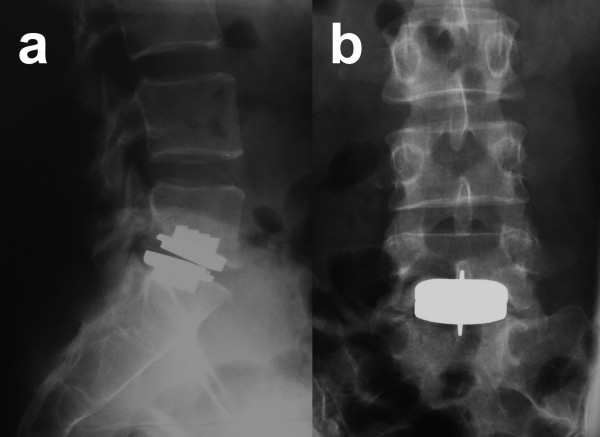
**A 42-year old female patient with degenerative disc disease received implantation of an artificial disc at L5-S1 (Prodisc, Synthes)**. Postoperative acute bilateral S1-pain without motor deficit could be explained by posterior dislocation of the inferior plate and a subsidence of the cranial plate of the disc prosthesis (a, b). This could clearly be visualised on plain radiographs. No dislocation of the PE inlay occurred which can be seen in the radiographic marker, being still in place. This patient was treated with a conversion into anterior fusion at L5-S1 with pain relief at the 1 year follow-up.

Daly et al presented a case series of five anterior dislocations of the Charité (Link) or the AcroDisc (DePuy) disc prosthesis causing compression and erosion of vascular structures. In figure 1 a case with a posterior dislocation of the inferior plate of an artificial disc is presented.

Mobile polyethylene cores can dislocate, too, and can cause compression to anterior abdominal or pelvic structures. This is especially true in non-constrained PE inlays (i.e. Charité TDR) Mathew et al [22] presented a case of an anterior polyethylene dislocation after lumbar TDR (Charité), which was then revised and fused with an anterior standalone cage. Within the case series by Kurtz et al [23] were 2 further cases and in the series of Leary et al [16] one case of anterior core dislocations. This complication required in all cases anterior PE core extraction and fusion. Reposition or exchange of the PE core alone is not recommended due to the prevalent biomechanical failure of the implant with risk of recurrence of dislocation.

Implant dislocation can easily be diagnosed with plain radiographs (figure 1). This is also true for PE core dislocations, since the PE-core contains dense markers in the available lumbar implants.

#### d) Implant failure/fracture

The long-term investigation of the first implanted lumbar discs (n = 53, mean follow-up 17.3 years; Charité, Link) by Putzier et al [24] included 7 implant fractures resulting in segmental fusion, all of which occurred with the earlier Charité II-model. Of these only one case underwent surgical revision requiring secondary fusion due to intractable pain. These cases show that the chosen implant has to stand a continuous load, requiring enduring metal alloys and stable implant design to provide a lasting product.

Implant fractures are mostly visible on plain radiographs.

#### e) Implant loosening/wear

Since implant wear has for decades been a major issue in hip and knee-arthroplasty it is not surprising that other weight-bearing implants with articulating surfaces – as in lumbar disc prostheses – will have to deal with implant wear and loosening, too. Punt et al [25] retrieved 16 artificial discs (SB Charité III, DePuy) which where explanted due to intractable pain after a mean of 8 years. They found polyethylene (PE)-wear debris causing chronic inflammation due to mononuclear macrophage and giant-cell activation. Kurtz et al [26] investigated 38 artificial discs (SB Charité III, DePuy) that were explanted in patients with therapy-resistant chronic pain after an average of 7.3 years (range 2.0–16.1). They found a positive correlation between PE inlay dome penetration and implantation time (r = 0.46, p = 0.004) and an average penetration rate of 0.5 mm/year (range 0.01–0.18 mm/year). These results could be validated in a PE-wear in vitro model [26].

Metal-metal articulations are known to cause metallosis with chronic inflammation leading to implant loosening [27]. François et al [28] presented a case of a loosened lumbar disc prosthesis with metal-metal combination (Maverick, Medtronic) one year after implantation causing persistent pain. They found in situ a loosening of the superior endplate and histologically typical signs of metallosis in the surrounding scar tissue. Cavanaugh et al [29] found similar hypergranulation patterns without histological metallosis surrounding a cervical explanted artificial disc causing re-stenosis 6 months after implantation. Implant loosening due to PE or metal wear is a known problem in TDR. Unfortunately not only loosening, but also symptomatic re-stenosis due to granulation caused by PE debris or metallosis were found.

Diagnostics of implant loosening range from standard plain radiographs – revealing radiolucencies around the implant – to scintigraphy and PET, showing increased metabolism in the bone-implant interface.

#### f) Central or lateral stenosis due to facet degeneration

**Figure 2:**
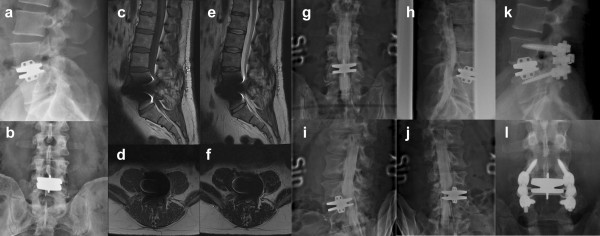
**46-year old male patient with increasing bilateral sensomotor deficit at L5 one year postoperatively after implantation of a metal-on-metal stainless steel artificial disc (Maverick, Medtronic) at L4–L5 (a, b) presented major artifacts on MR imaging in T1 (c, d) and T2 (e, f)**. Neurophysiological investigations revealed acute bilateral L5-compression. Leading diagnostic procedure that could visualise a bilateral compression of L5 was plain myelography, which revealed an anterior compression of the thecal sac (g, h) and compression of the right (i) and left (j) L5-root. Therefore a posterior decompression and instrumented posterolateral fusion was performed while keeping the implant in situ (k, l). Intraoperatively facet hypertrophy and posterior bulging of remaining annulus was seen. The sensomotor deficit resolved completely. The patient regained function and returned to work.

The case in figure 2 is a typical example of bi-radicular stenosis after disc replacement. Due to remaining annulus a progressive degeneration of the facet joints most likely caused stenosis leading to nerve root compression one year postoperatively. Postoperative pain has been described in long term studies, and some authors relate this to facet degeneration to hyperlordosation stressing the importance of implant positioning on patient outcome [30]. Park et al [31] found in about 30% of the cases progressive facet degeneration, which was more common in women, malposition of the implant in the frontal plane and multilevel TDR.

MR diagnostics are the gold-standard for investigation of spinal canal pathologies. In cases where this is not possible – i.e. stainless steel TDR – other modalities as CT and myelography have to be considered.

#### g) Implant malpositioning

Bendo et al [32] found the midline retroperitoneal access associated with less sagittal implant misplacements than the pararectal lateral retroperitoneal access (p = 0.021), but this did not have any impact on clinical functional outcome, determined with the Oswestry disability index (p = 0.92). Rauschmann et al [33] presented in a cadaver study improved positioning of the lumbar artificial disc if navigation was applied compared to fluoroscopy (n = 30). Malpositioning can be due to minimal malrotation of the image intensifier, anomalities in the spinal process anatomy, and the parallax effect in intraoperative fluoroscopy [34]. Navigation can therefore have significant impact on the quality and durability of artificial discs in the future.

To control implant positioning plain radiographs are usually sufficient.

#### h) Segmental ankylosis

Long-term investigations failed to present preservation of motion of the operated segment. Putzier et al [24] found that 60% of all treated patients (n = 53) had spontaneous fusion of the treated segment after a mean follow-up of 17-years (Charité, Link). Interestingly these patients were better than those with remaining motion with regard to functional measures (ODI, p < 0.05) and pain (VAS, p < 0.05). Thus fusion should be viewed as a natural course of motion preservation treatment and not as a complication.

To diagnose fusion either a CT-scan showing ankylosis or dynamic radiographs excluding remaining segmental mobility are required.

#### i) Progressive implant subsidence

In osteoporosis or poor bone quality, years after the original implantation, the artificial disc can subside into the endplates, causing secondary implant dislocation and increasing pain. To avoid this complication the choice of the largest possible implant and a placement close to the rim of the endplates is recommended [30].

To visualise implant subsidence plain radiographs are sufficient. If an osteoporotic vertebral endplate fracture is suspected, radionuclide scans may show increased uptake of the fractured vertebra.

## Conclusion

Postoperative imaging of lumbar total disc replacement requires alternative imaging techniques to conventional MRI-diagnostics. All currently available lumbar artificial discs contain stainless steel alloys, and, therefore, generate major artefacts on MRI. Alternative imaging modalities include plain film radiographs, myelography and radionucleotide imaging. These imaging modalities should be utilised in cases with suspected postoperative complications after lumbar TDR.

The appearance of artificial discs on the implant market meant a drawback in postoperative radiological transparency with regard to MR-diagnostics, since all lumbar disc prostheses are still based on stainless steel alloys, thus causing major artefacts. Other imaging modalities are available and are in the first line of choice in cases with suspected postoperative complications after lumbar TDR. Until lumbar titanium artificial discs are available postoperative imaging of the operated segment requires altered radiological strategies.

## Competing interests

The authors have no competing interests with regard to the publication of this manuscript.

## Authors' contributions

YR wrote the manuscript and BS critically revised it. Both authors read and approved the final version of the manuscript
